# 2689. Utility of Bronchoalveolar Lavage for Diagnosis and Management of Pneumonia in Immunocompromised Children

**DOI:** 10.1093/ofid/ofad500.2300

**Published:** 2023-11-27

**Authors:** Amanda M Green, Tim Flerlage, Diego Hijano, Afreen Abraham

**Affiliations:** St Jude Children's Research Institute, Memphis, Tennessee; St Jude Children's Research Hospital, Memphis, Tennessee; St. Jude Children's Research Hospital, Memphis, Tennessee; St Jude Children's Research Hospital, Memphis, Tennessee

## Abstract

**Background:**

Determining the etiology of acute respiratory illness (ARI) in immunocompromised children is challenging. The wide variety of infectious and non-infectious causes makes it difficult to optimize empiric antimicrobial treatments, leading to broad, lengthy antimicrobial courses with potential antimicrobial overuse, increased side effects, and interruptions to chemotherapy treatments. The diagnostic yield and safety of bronchoalveolar lavage (BAL) in these patients has long been debated.

**Methods:**

We conducted a retrospective chart review of BAL procedures performed on immunocompromised children with ARI at St. Jude Children’s Research Hospital between 2017-2021. Microbiological and molecular study results were reviewed, including next generation sequencing (NGS) studies obtained from BAL, upper respiratory, and blood specimens within 7 days of the BAL. Etiology determined by the clinical team, changes in antimicrobial clinical management, and adverse events following the BAL were collected.

**Results:**

82 BAL procedures from 57 patients are presented. The most common underlying diagnosis was hematologic malignancy, and 39 (48%) had undergone hematopoietic cell transplant. BAL identified one or more potential respiratory pathogen in 62.2% of ARIs, including viruses (54%), bacteria (48%), and fungi (37%). Of the 12 patients with BAL NGS, 8 (67%) identified likely respiratory pathogens, 5 (42%) correlated with other testing, and 3 (25%) were unique to NGS. 58 (71%) of BALs led to a change in antimicrobial management, either by discontinuation of broad-spectrum antimicrobials (65%) or narrowed antimicrobial regimen (41%). In 7 (12%) patients with suspected infectious etiology, BAL confirmed alternate etiology and avoided initiation of antimicrobials. 12 (15%) of patients underwent new intubation, and 22 (27%) had increased ventilatory requirements within 24 hours of BAL. No patients required new ICU transfer due to the procedure.

**Figure:**
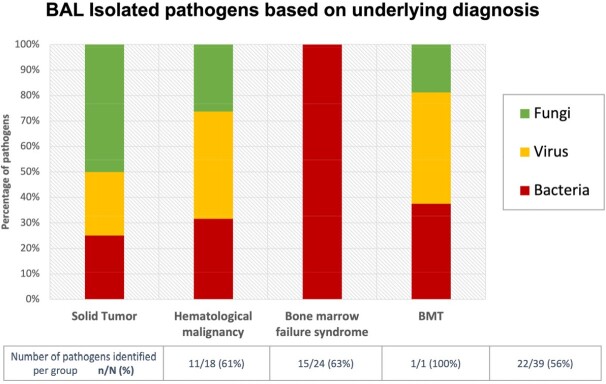

**Figure:**
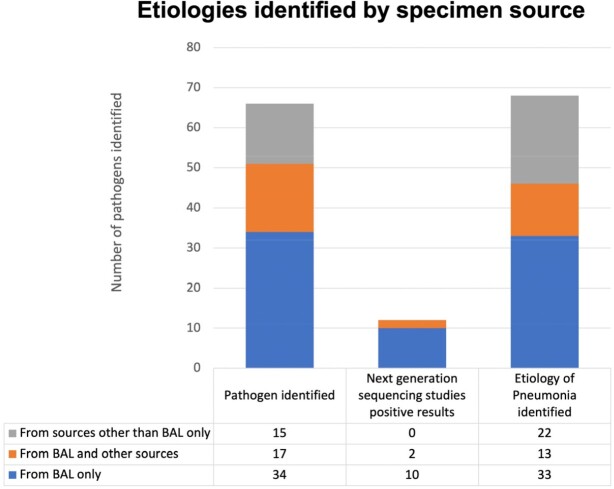

**Conclusion:**

BAL is a valuable, well tolerated procedure that can aid in the diagnosis of pulmonary disease in pediatric immunocompromised patients. These preliminary findings highlight the benefits of BAL and its potential impact on decision making and clinical management, including avoiding antimicrobial overuse.

**Disclosures:**

**All Authors**: No reported disclosures

